# Improving Patient Safety in General Hospitals Using Structured Handoffs: Outcomes From a National Project

**DOI:** 10.3389/fpubh.2022.777678

**Published:** 2022-03-16

**Authors:** Orly Toren, Michal Lipschuetz, Arielle Lehmann, Gil Regev, Dana Arad

**Affiliations:** ^1^Patient Safety and Risk Management, Hadassah-Hebrew University Medical Center, Jerusalem, Israel; ^2^Nursing Department, Ono Academic College, Kiryat Ono, Israel; ^3^Psyfas, Teamwork and Healthcare, Herzliya, Israel; ^4^Patient Safety Division, The Israeli Ministry of Health, Jerusalem, Israel

**Keywords:** handoffs, team communication, ISBAR, patient safety, standardized tool, patient's transfer

## Abstract

**Background:**

Promoting quality and patient safety is one of the health policy pillars of Israel's Ministry of Health. Communication among healthcare professionals is of utmost importance and can be improved using a standardized, well-known handoff tool such as the Introduction, Situation, Background, Assessment, and Recommendations (ISBAR). This study aims to present implementation process and participants' satisfaction of a national project that used a standardized tool for team communication.

**Methods:**

This national intervention project included process implementation teams from 17 Israeli general hospitals evaluating the ISBAR implementation process for transferring patients from intensive care units to medical/surgical wards. The project, conducted between January 2017 and March 2018, used Fischer's test and logistic regression. The project evaluation was based on the participants' assessment of and satisfaction with the handoff process.

**Results:**

Eighty-seven process implementers completed the questionnaire. A statistically significant increase in satisfaction scores in terms of four variables (*p* < 0.001) was observed following the implementation of the project. Nurses reported higher satisfaction at the end of the process (0.036). Participants who perceived less missing information during handoffs were more satisfied with the process of information flow between wards (84.9%) than those who perceived more missing information (15.6%). Participants who responded that there was no need to improve information flow were more satisfied with the project information flow (95.6%) compared to the group which responded that it was necessary to improve information flow (58.2%). Three out of four variables predicted satisfaction with the process. Being a nurse also predicted satisfaction with information flow with a point estimate of 2.4. The C value of the total model was 0.87.

**Conclusions:**

Implementation of a safety project at a national level requires careful planning and the close involvement of the participating teams. A standardized instrument, a well-defined process, and external controls to monitor and manage the project are essential for success. Disparities found in the responses of nurses vs. physicians suggest the need for a different approach for each profession in planning and executing a similar project in the future.

## Introduction

One of the main goals of the Israeli Ministry of Health (MOH) is to promote efforts to improve quality and safety in the healthcare system. Approaches to achieving this goal include implementing patient safety programs and developing policies and procedures for the prevention of adverse events, as well as identifying areas of weakness and initiating possible solutions (1). A major area of weakness identified by risk managers is communication between departments (2, 3).

National safety projects can be an effective platform for implementing change. Communication between teams is an integral part of patient care and is of paramount importance in ensuring patient safety and has a positive impact on handoff communication by the staff (4–6). Suboptimal communication between health care providers is a common issue and may result in medical errors (7, 8) and medical malpractice lawsuits (9, 10). Therefore, effective communication between health care providers is essential for ensuring safety and quality of care (11).

When patients are transferred from one unit to another, it is vitally important to communicate necessary patient information to ensure continuity of care (8). The potential for the transfer of incorrect information, or for information to be missing constitutes a safety hazard (5, 6). While a patient transfer is usually accompanied by verbal handover in the form of a unstructured conversation, that conversation actually contains highly important information required for effective continuity of care (12). Unfortunately, communication failures during patient transfers are widespread and can lead to delays in diagnosis and treatment and to adverse events (13, 14).

In 2006, the International Joint Commission identified the need for structured communication during patient transfers and defined requirements for the use of a structured tool (15). Other organizations, including the World Health Organization, the Australian Commission for Safety and Quality in Health Care, and the Society of Hospital Medicine, also adopted this recommendation for patient transfers within hospitals (16–18).

According to this recommendation, transfers of patients between health care practitioners should emphasize specific relevant information, separate from unimportant data, and should avoid subjective interpretations. The quality of the information has vital repercussions for the decision-making process and the delivery of proper care (14). The use of a structured communication format in patient handoffs helps professionals focus on the important details and minimizes the likelihood of errors (19, 20).

Two factors are of critical importance when discussing communication and patient handoffs. The first is the tool that is used. Most of the current literature concerning the issue of communication and patient handoffs focuses on transferring patients between wards using a communication instrument tailored to the specific clinical field or hospital unit, and therefore, the generalizability of the findings from these studies is limited. The second factor is the setting. Many studies have focused on the association between handoffs during shift changes within the ward and patient outcomes (13, 21, 22). However, recent literature shows a shift in focus to interdepartmental communication when patients are transferred between wards, units, or health care institutions. Interdepartmental communication resulted in a perceived improvement in the quality of verbal handoffs (6).

This quality-improvement study aimed to improve interface communication between intensive care units and general wards (medical and surgical) by providing a structured communication tool tailored specifically to their handoff needs. To our knowledge, this is the first national quality improvement project that describes the process of creating and implementing a communication handoff tool among the majority of hospitals in Israel and measures the participants' evaluation of and satisfaction with the process. The aims of the project were: to implement a standardized communication transfer tool in all the general hospitals in Israel; to assimilate continuous active peer learning throughout the project to optimize the implementation strategies; to examine and assess different aspects of the project components, including the implementation process, team involvement, similarities and differences between units and health care providers, and the tool itself; and to evaluate overall team satisfaction.

This article presents the project's stages (planning, implementation, and evaluation) and draws conclusions for suggested national policies and regulations.

## Methods

### Context

Effective communication between healthcare providers is an essential safety factor for preventing errors. Therefore, in order to identify main sources of interdepartmental communication errors, we conducted a survey among risk managers in Israeli hospitals. The results of the survey indicated that communication errors occur primarily during the interfaces between intensive-care units (ICUs) and general (medical/surgical) wards and between emergency departments and in-patient wards.

Transferring a patient from the ICU to a medical or surgical ward and vice versa presents unique challenges for both teams. Generally, the patient is accompanied to the receiving ward by one or more healthcare personnel, has extensive, complex monitoring equipment, and has been prescribed various medications. In the receiving ward, multiple activities, such as replacing equipment and delivering clinical information to the receiving team, occurred simultaneously upon patient arrival while the patient is monitored. This process often takes place in a chaotic and busy environment, with a multidisciplinary team with diverse experience and often with subjective interpretations transferring the information. Under such circumstances, handoffs between teams need to be concise and efficient (14).

We decided to focus this study on the interface between ICUs and general wards because of the critical importance of the communication process during patient transfers between these departments and because these patient handoffs are more defined and less complex than handoffs from the emergency department to general departments. Furthermore, it was assumed that this interface had a higher potential for successful implementation and adaptation of the process.

The project was initiated by the MOH and was managed as a quality improvement project, as routinely performed by the MOH. The intervention took place from January 2017 through March 2018. Ono Academic College in Kiryat Ono, Israel, provided ethical approval of the study (Trial Ethical Approval Number: 202131ono) and waived the requirement for informed consent from participants because this was a quality improvement project.

### Study Population

Hospitals included in the study were general hospitals with at least one surgical or medical intensive care unit and at least one medical or surgical ward. Electronic questionnaires were sent to participants via email using a specific link. The questionnaires stated that the data collected would be used for research purposes. All questionnaires and data were collected anonymously. Filling out the questionnaire was deemed to constitute consent to participate in the study. Participation in the project was voluntary. Of 23 eligible hospitals, 17 hospitals participated (74% response rate). There were no conflicts of interest.

Three departments (ICU, surgical ward, and medical ward) selected from each of the 17 hospitals were included in the study. A senior member from the risk management department at each hospital was appointed to coordinate and implement the project at the hospital. Risk managers from participating hospitals verbally consented to participate in the project. A physician and a nurse from each ward or unit were appointed to guide and implement the process in their respective units. Overall, the study included 102 participants: 51 physicians and 51 nurses.

### Intervention

The ISBAR instrument is a communication tool designed to contain the pertinent information needed to be conveyed during an emergency: introduction (self-presentation of the delivering and admitting team members); situation (what is happening here and now; delivering the facts); background (the patient's relevant medical and psychosocial history and background diseases); and assessment (trend assessment). This tool was chosen for the study because it is a recognized method for conveying vital information under time constraints and in high-risk environments such as ICUs (23). The ISBAR method structures communication so that concise and essential information required for treatment and decision-making is delivered. The use of this method promotes treatment safety and quality and minimizes errors (24, 25).

Relevant content to be documented and delivered during patient handoffs was formulated during a number of meetings with team leaders, process implementers, representatives from the Ministry of Health's safety unit, and accompanying consultants. A uniform ISBAR format was initially developed, and during subsequent deliberations, a slightly broader ISBAR format was formulated to better suit complicated patients hospitalized in ICUs. The finalized format maintained the required objectives. During a simulation before the initial use, the transfer of information took ~1–2 min.

### Structured Supervision and Project Evaluation

Intensive training was provided by the hospital project leaders (head nurses and senior physicians) during simulation workshops for physicians and nurses in all participating units and departments.

The implementation process, including satisfaction with the project implementation, were evaluated by the project implementers before, during, and at the end of the process. The questionnaire was based on the ISBAR principles of communication of vital information in stressful/high risk environments. An expert content validation of the relevance of the questionnaire and a modification were conducted during the process. Upon completion of this process, the short questionnaire tool was designed. The questionnaire evaluated four aspects of the project leaders' perceptions: (1) problems with information flow–such as lack of significant information during handoff; (2) the need to improve information flow; (3) frequency of communication errors resulting in incorrect information flow; and (4) the use of a uniform format for data during patient transfer. A question of overall satisfaction with the project was included in the questionnaire. Answers were rated on a 4-point Likert scale (1 = low, 4 = high). At the ward level, different tools were created for nurses and physicians for use prior to and during the transfer. The adaptations were based on these specific tools to monitor the transfer process. Based on this evaluation, the supervisors were able to monitor performance in each hospital and to make overall improvements during the process. A meeting of all project leaders, MOH representatives, and external consultants took place on a quarterly basis. The agenda for each meeting included a report on the project's progress at each hospital and a discussion regarding problems that had arisen. Some problems were common to all the hospitals, and a uniform procedure for addressing these issues was established. During the meetings, solutions were offered for dealing with difficulties that some sites faced, and techniques for optimizing and maintaining the process (such as forms, endurable working processes, and data transfer) were discussed.

External consultants were available to answer questions and to respond to difficulties that arose. Weekly contact with each hospital leader was maintained to discuss implementation issues during handoffs. In addition, the consultants visited each of the participating hospitals 2 to 3 times during the course of the project.

Nurses and physicians were supervised by project leaders throughout the year-long project implementation period. However, throughout the year, nurses adhered more closely to the supervision process than was initially expected, while physicians' cooperation with supervision was less sustained as the project progressed.

#### Project Implementation

Implementation at the hospital level was led by the project leaders: a physician and a nurse, typically a head nurse and a senior physician. In all hospitals, discussions were held between the staff of the ICU and the wards concerning the optimal work process to facilitate the most efficient communication. In many hospitals, the implementers created guidance and training materials, such as patient transfer videos, using the ISBAR method.

Although the project was essentially multisectoral and intended for collaborating teams of physician and nurses, it was determined that communication concerning patient transfers would take place separately within the nursing and the physician teams, in light of differing conditions in the units and the requests of staff. This required planning and the implementation of the handoff process between both the nurse teams and the physician teams before transferring patients from the ICU to the ward and after admission.

##### Training Teams

A meeting with the hospital's senior management was held to introduce the project in each hospital. Subsequently, the project was presented to every department during a team meeting. This meeting consisted of describing the project, customizing work processes, and presenting the ISBAR tool. In some hospitals, additional training was conducted using simulations.

##### Handoff Instruments

To facilitate ease of use, the ISBAR tool was designed in 2 formats: (1) a pocket-card format, using a card attached to employees' identification card, making it more available during handoffs, and (2) a poster format (A4 size), posted at work stations next to computers and telephones as a constant reminder and resource that was available to the teams.

In addition, a supervision assessment tool to evaluate detailed information transferred orally was used by the admitting department and occasionally by the receiving department. This tool was kept in a special folder within each department and was used to evaluate whether required information from each of the parameters listed in the ISBAR had been communicated. At the beginning of the process, the assessment was carried out by the head nurses together with the project leaders of the physicians. However, because of a rapid learning curve, the supervision assessment was soon carried out solely by the head nurses given their greater cooperation with the supervision process, as opposed to the physicians. When disparities in information were discovered, the issue was examined with both the ICU nurse and the ward nurse during the team meetings.

### Satisfaction Evaluation

Project evaluation was based on a questionnaire that evaluated aspects of information flow experienced in the ISBAR process as well as overall satisfaction with the project. The questionnaire was modified based on expert content validation during the process by the project leaders. The questionnaire measured four areas assessing the quality of communication specifically related to patient transfers between the departments–lack of information during handoff; the need to improve information flow; frequency of communication errors, and the use of a uniform format during patient transfer. Overall satisfaction from the transfer communication process was also measured. The questionnaire was clear to the users and easy to use. It was sent digitally to all implementers on 3 occasions: before the project's implementation, 6 months after the project's initiation, and at the end of the program. Responses were rated on a 4-point Likert scale ranging from 1 (*very low*) to 4 (*very high*). The data were aggregated into 2 groups: responses of 1–2 (*very low* or *low*) and 3–4 (*high* or *very high*).

### Statistical Analysis

We used multiple methods to analyze the data. During the implementation phase, we held a monthly peer learning meeting that included discussions and sharing of knowledge regarding challenges during implementation in the hospitals, such as refusal to participate or refusal to document the data transferred. We used qualitative methods to draw inferences from the data discussed.

The satisfaction questionnaires were analyzed using the Statistical Package for the Social Sciences (SPSS), English version 24. Frequencies and descriptive statistics were used to describe sample demographics. Questions phrased negatively were reversed for analysis. Fisher's exact tests were used to test the significance of each component in the questionnaire. Differences between the variables ranged over time were evaluated using a chi-square test and univariate analysis was used to examine correlations among variables. A logistic regression was performed using a model including all the variables that were found to be significantly correlated with satisfaction.

## Results

A total of 87 process implementers completed the questionnaire before initiation of the project (85% response rate), and 50 (49% response rate) completed the questionnaire at the conclusion of the project (~1 year after its initiation).

A statistically significant increase in satisfaction scores was observed in all questionnaire aspects examined before and after implementation (Table 1). At the end of the project, fewer team members reported missing significant information during patient handoffs or needing to improve data flow. In addition, there was a lower frequency of communication errors, and a greater number of team members reporting using a uniform communication format during patient transfers. There was also significant improvement in satisfaction with the process of information flow between wards when comparing satisfaction before implementation and at the end of the project (Table 1).

**Table 1:** Evaluation of communication before and after project implementation.

**^**a**^**	**Before implementation, No**.	**During implementation**,	**After implementation**,	***P* value^**b**^**
	**(%) (*n* = 87)**	**No. (%), (*n*-78)**	**No. (%) (n= 50)**	***(Before vs. After)***
Lack of significant information during handoff	31.8 (27)	5.2 (4)	2.0 (1)	<0.001
The need to improve information flow	79.3 (69)	38.2 (29)	48.0 (24)	<0.001
Frequency of communication errors	43 (37)	10.5 (8)	12.0 (6)	<0.001
Use of a uniform format for data during patient transfer from unit to ward	39.5 (34)	83.3 (65)	72.0 (36)	<0.001
Satisfaction with the process of information flow between wards	56.3 (49)	89.7 (70)	82.0 (41)	0.002

a *The number of high or very high responses is provided for each question*.

b *Fisher's exact test between responses of very low/low and high/very high*.

Differences between physicians' and nurses' satisfaction with the program could not be analyzed owing to the limited number of responders at the end of the process (45), of which only 12 were physicians.

Table 2 describes the distribution of satisfaction (responses of *high* or *very high*) of physicians and nurses before and at the conclusion of the project. Nurses reported higher satisfaction at the end of the program in nearly all parameters studied compared with the initiation of the program. Statistically significant improvement in satisfaction was noted in all aspects other than interdepartmental information flow, where the change noted nearly reached statistical significance (*P* = 0.06).

**Table 2:** Comparison between physicians and nurses who indicated high or very high project satisfaction before and after project implementation.

**Question**	**Profession**	**Before implementation, No. (%)**	**During implementation, No. (%)**	**After implementation, No. (%)**	***P* value^**a**^**
		***n* = 45 nurses, *n* = 36 physicians**	***n* = 58 nurses, *n* = 19 physicians**	***n* = 35 nurses, *n* = 15 physicians**	
Lack of significant information during handoff	Nurses	20.9 (9)	1.8 (1)	0	0.004
	Physicians	38.9 (14)	15.8 (3)	6.7 (1)	0.021
The need to improve information flow	Nurses	75.6 (34)	36.8 (21)	45.7 (16)	0.006
	Physicians	83.3 (30)	38.9 (7)	53.3 (8)	0.025
Frequency of communication errors	Nurses	44.4 (20)	8.5 (5)	8.6 (3)	<0.001
	Physicians	40 (14)	16.7 (3)	20 (3)	0.177
Use of a uniform format for data during patient transfer from unit to ward	Nurses	50 (22)	86.2 (50)	88.6 (31)	<0.001
	Physicians	25 (9)	73.7 (14)	33.3 (5)	0.545
Satisfaction with the process of information flow between wards	Nurses	68.9 (31)	94.8 (55)	88.6 (31)	0.036
	Physicians	44.4 (16)	73.7 (14)	66.7 (10)	0.147

a *Based on a Fisher's exact test comparing responses before and after project implementation*.

Among physicians, the findings pointed to a similar trend; however, the changes between the commencement of the program and its end did not reach statistical significance, likely owing to the small number of physician responders (36 at the beginning and 12 at the end). The increase in satisfaction level regarding missing significant information during patient handoffs, as evaluated by physicians before and after the project, nearly reached statistical significance (*P* = 0.07), although the number of respondents was limited (Table 2).

Since the sample in each period was too small for correlation analyses, a total of the complete sample was used to analyze correlations between the variables (*n* = 215). The variables used for analysis were coded for high (high or very high in the questionnaire) and for low (low or very low in the questionnaire). Table 3 describes the distribution of all respondents during the year of implementation in terms of the different variables correlated with satisfaction with the process of information flow (*n* = 215). All the variables related to the ISBAR process were significantly correlated with high satisfaction. Participants who perceived low levels of missing information during handoffs were more satisfied with the process of information flow between wards (84.9%) than those who perceived high levels of missing information (15.6%). Participants who responded that there was no need to improve information were more satisfied with the project information flow (95.6%) compared to the group that responded that it was necessary to improve information flow (58.2%). Participants who said that communication errors are infrequent were more satisfied with the project (85.1%) than those who perceived frequent communication errors (41.2%). Those who experienced the transfer with an existing format were more satisfied (87.4%) than those who responded that there was no uniform format involved during transfer (51.9%). The project period was correlated with satisfaction from the second phase of measurement. Nurses were more satisfied than physicians (84.8 vs. 57.1%). No significant difference in project satisfaction was found between hospitals of different sizes or between different types of ward.

**Table 3:** Distribution of all respondents during the year of implementation among the various variables correlated with the project satisfaction (*n* = 215).

**Question**	**Responses^**a**^**	***N***	**High satisfaction^**b**^**
Lack of significant information during handoff^*^	Low	179	84.9%
	High	32	15.6%
The need to improve information flow^*^	Unnecessary	91	95.6%
	Necessary	122	58.2%
Frequency of communication errors^*^	Uncommon	161	85.1%
	Common	51	41.2%
Use of a uniform format for data during patient transfer from unit to ward^*^	No format exists	79	51.9%
	Format exists	135	87.4%
Type of unit	ICU	64	75.0%
	Internal department	80	77.5%
	Surgery department	51	76.5%
Period^*^	Before	87	56.3%
	During	78	89.7%
	After	50	82.0%
Role^*^	Nurse	138	84.8%
	Physician	70	57.1%
Hospital size	Small (<400 beds)	113	71.7%
	Medium (400–800 beds)	53	71.7%
	Large (>800 beds)	44	84.1%

a *The number of high or very high responses vs. Low and very low is provided for each question*.

b *The number of high or very high satisfaction*.

* *P < 0.05*.

To predict satisfaction with information flow using the ISBAR process, a logistic regression was used, entering all four variables concerned, in addition to role (nurse/physician) and study period. Since hospital size and type of unit were not significant in the univariate model, they were removed from the equation. As shown in Table 4, similar to the correlation analysis, three of the four items of the ISBAR process were significant in predicting satisfaction (except frequency of communication errors). The only other variable that predicts satisfaction with information flow was being a nurse, with a point estimate of 2.4 for nurses as opposed to physicians. The C value of the total presented model was 0.87.

**Table 4:** Logistic regression model predicting satisfaction using the ISBAR.

**Variables**	**BETA**	**SE**	**Wald Chi-Square**	***P*-value**	**OR**	**CI**	
Nurse	0.8779	0.4572	3.6876	0.0548	2.406	0.982	5.894
Lack information	−2.0613	0.7000	8.6713	0.0032	0.127	0.032	0.502
Improve information flow	−1.6867	0.6154	7.5125	0.0061	0.185	0.055	0.618
Uniform format	1.0424	0.4744	4.8275	0.028	2.836	1.119	7.187
Period (after vs. before)	0.049	0.57	1.54	0.46	1.05	0.34	3.21
Period (during vs. before)	0.68	0.57	154	0.46	1.92	0.62	6.17
Frequency of communication errors	−0.22	0.56	0.15	0.69	0.80	0.26	2.44

## Discussion

In this national quality improvement project, we aimed to improve patients' safety by implementing a standardized handoff tool to improve communication between medical teams while transferring patients between ICUs and general hospital departments (medical or surgical).

Such a project at a national level requires careful planning and close involvement of the participating teams. The success of the project was based on several elements, including a well-defined process, external control to monitor and manage the project, real-time problem-solving using peer learning, and teams' adherence to the project. The disparities between nurses and physicians suggested the need for a different approach for each profession in planning and executing similar projects in the future.

### Interpretation Within the Context of the Wider Literature

Patient handoffs within hospital departments and to other health care facilities are a potential weak point in patient safety and require special attention. Professional experience, as well as the published literature, indicate that vital and, at times, critical information affecting patients' wellbeing may be omitted during patient transfer (4, 6–8, 13, 21, 26). To maintain continuity of care, a standardized and structured communication format during handoffs is needed, in accordance with the International Joint Commission's accreditation requirements (15). Thus far, attempts to improve interdepartmental communication have focused on specific departments and on developing unique tools for these departments (14, 17, 24). This article presents an implementation of a wide-scale national project for patient handoffs between ICUs and medical/surgical wards using a uniform, standardized tool. The project included 17 hospitals throughout Israel, representing 74% of the 23 hospitals that met the project's inclusion criteria, allowing the results to be generalized to all general hospitals in the country.

At the assessment after project completion, the process implementers reported increased satisfaction in all the aspects measured. They reported a reduced incidence of missing significant information during handoffs, a decreased need to improve information flow, fewer communication errors, greater use of uniform forms during handoffs, and more satisfaction with the information flow between hospital departments. These trends were consistent with reports from the hospital leaders and indicate that the process may have contributed to improved patient safety.

Both physicians and nurses expressed increased satisfaction at the conclusion of the project, but the change in satisfaction from the start to the end of the program was found to be statistically significant only among the nurses. These results are consistent with reports received from hospital leaders during the project, in which nurses, more than physicians, raised the need to improve communication between departments and consequently fully cooperated to promote the project throughout the implementation process. These findings may also be attributable to a decline in the physicians' response rate at the end of the program and to the steady attrition rate of physicians in the regression analysis. Only being a nurse predicted satisfaction with information flow between wards.

Among physicians, there was a sense that communication during patient handoffs does not directly affect patient safety. Efforts were made to explain to the physicians that standardizing the patient handoff process has a positive effect on patients' safety. The subjective evaluations of the hospitals' leaders suggested that physicians expressed concern regarding the suitability of the project and questioned whether it added value to an existing process that seemed to them adequate and safe. Some physicians withdrew from the project owing to their belief that it did not benefit their routinely performed handoff process. Differences in physicians' responses to the project call for additional consideration concerning implementation within this group.

Communication based on the ISBAR format distinguishes between essential and nonessential information and is meant to contribute to assisting both physicians and the nursing staff. The differences between the responses from nurses and physicians may be related to the differences in their work processes. For example, differences between them may stem from nurses being more accustomed to methodical work based on protocols than are physicians. Physicians reported that filling out the ISBAR along with all the other documentation was difficult and constituted redundant paperwork. Nevertheless, in the ISBAR training simulations held with physicians, it was repeatedly found that without proper documentation like that in the ISBAR format, important medical information was omitted. For this reason, we believe that the implementation of the process should continue among physicians as well, while at the same time realizing that a conceptual shift in the work culture may be required. Such a conceptual shift will enhance the implementation process and relevance to the sector (27).

Apart from role (being a nurse or a physician) all 4 aspects of the ISBAR examined in this projects were associated with project satisfaction. i.e., lack of significant information during handoff, the need to improve information flow, frequency of communication errors and the use of uniform format during patient transfer, and period of the implementation during and at the end of the project. This is consistent with other studies evaluating the impact of implementation of standardized handoff tools (21). However, a standardized tool may not be sufficient to prevent information decay in complex care units such as ICUs (28). To overcome this limitation, our tool's validity was evaluated by project leaders who represented the needs of the ICU, surgical, and medical wards. Hospital size and type of ward did not correlate with satisfaction in our study, and we did not find other studies with such correlations in the literature. However, when predicting satisfaction with all the variables that were found to be correlated with satisfaction, only the role of nurse and three other variables were meaningful in terms of significant information shortfalls during handoff,– the need to improve information flow, the use of uniform formats during patient transfer, and the period of the implementation during and at the end of the project (29). Surprisingly, frequency of communication errors was not found to be a significant predictor of satisfaction. This might be related to the subjective way errors were measured and due to recall bias. Another explanation could relate to cognitive informatics (27).

### Implications for Policy, Practice, and Research

#### Project Expansion on a National Level

It is feasible to expand this project to the national level, based on the policy of the MOH. A project of this magnitude requires careful planning and extensive knowledge of all the organizations involved in the project.

#### Accepting the Change

Overall, the implementation of the change was easier among the nursing staff than with the physicians. A policy aimed at promoting safety and focusing on team communication should take into account the differences between the nursing and physician teams and determine the appropriate intervention for each team.

#### Changes in Work Processes to Increase Safety

As part of the project goals, the teams examined work processes and identified junctures that were potential safety threats. Therefore, in the majority of hospitals taking part in the project, the rate of transfers from the ICUs to the wards increased during morning shifts in comparison with other shifts. This change is highly desirable and contributes to safety, because more senior clinicians are present during the morning hours, whereas other shifts are staffed by fewer and less-experienced physicians.

The information transferred during shift changes includes information related to patients awaiting transfer. As a result, this project raised staff awareness regarding these patients and their vulnerability. Physicians' responsibility was also discussed. A senior physician during the day shift or a specialized intern during the other shifts was chosen to transfer information using the ISBAR method to their counterpart in the receiving ward.

A uniform format used for communication facilitates better patient-specific preparation, thus enabling the receiving ward to prepare for a specific patient's health characteristics and to guarantee continuity of care.

#### Expanding the Scope

This method could be expanded beyond the departments included in the initial pilot program. Increased awareness in the participating departments and a shift to an active approach of retrieving ISBAR information was noted in most hospitals during handoffs between other participating units.

It should be noted that in the majority of hospitals, the process was expanded beyond the initial departments owing to the requests and needs of other units. This indicates a considerable intra-hospital need for optimizing the information flow during patient transfers and the suitability of the ISBAR method.

### Recommendations for Success

#### Promoting the Projects' Goals

For a project of this magnitude to succeed, attention must be given to making the staff aware of the project's necessity and to planning ahead to incorporate developments in various medical fields. Recommendations for the following steps stem from our experience on a national scale include:

- Selecting the types of departments for implementation and investing time to motivate teams about the importance of the method and the team members' vital role in the process.
- Selecting project leaders and creating a peer forum in which leaders can meet for the purpose of mutual learning and brainstorming.
- Selecting a method that suits the needs and characteristics of the hospital and its participating departments. The criteria on which planners should base the handoff method is that information is transferred in a concise, clear, and practical manner. The chosen ISBAR method in the current project meets these criteria.
- Selecting a tool and customizing it to all participating departments. The ISBAR format should be specifically customized for each interface, and the format should be shared with and agreed upon by both the transferring and the receiving teams. To this end, reaching a consensus between all the project leaders regarding the format and the implementation method is recommended.

#### Training Teams

New team members should be trained to deliver and admit patients using the ISBAR method. In this study, the training method that was proven most effective was a simulation in which feedback was provided.

#### Ongoing Maintenance

To maintain the process and guarantee its execution over time, supervision and observations concerning ISBAR deliverance are recommended.

### Study Limitations

To our knowledge, this is the first national project in Israel to promote patient safety based on a standardized communication tool for patient handoffs between hospital departments.

This large-scale project had limitations. The project evaluation represented the process implementers' perspectives, but included no objective measurements. In addition, the data on project satisfaction were collected from the process implementers only. Teams' views regarding the process, its impact on safety, and their satisfaction should be recorded in future research. Although a substantial number of hospitals were part of this project, the low response rate, specifically from the physicians, hampered the ability to draw comprehensive conclusions.

In addition, data were collected only until the end point of the project's implementation. To assess the long-term effects of the project, lengthier follow-up research is needed.

## Conclusions

A national project presents great advantages, including the ability to generalize the impact of patient safety to a larger part of the health care system. Implementation of a safety project at a national level requires careful planning and close involvement of the participating teams. Using a standardized instrument and a well-defined process, along with external control to monitor and manage the project, is necessary for success. Disparities between nurses and physicians necessitate a different approach for each profession in planning and executing a similar project in the future.

## Data Availability Statement

The original contributions presented in the study are included in the article/supplementary material, further inquiries can be directed to the corresponding author/s.

## Ethics Statement

The studies involving human participants were reviewed and approved by Ono Academic College in Kiryat Ono, Israel. Written informed consent from the patients/ participants was not required to participate in this study in accordance with the national legislation and the institutional requirements.

## Author Contributions

OT was a major contributor in leading the project, writing the manuscript, and interpreting data. ML was a major contributor in writing the manuscript. AL and GR were contributors in writing the manuscript. DA was a project manager initiator, contributed to data analysis, and writing the manuscript. All authors contributed to the article and approved the submitted version.

## Funding

This work was supported by the Israeli Ministry of Health.

## Conflict of Interest

The authors declare that the research was conducted in the absence of any commercial or financial relationships that could be construed as a potential conflict of interest.

## Publisher's Note

All claims expressed in this article are solely those of the authors and do not necessarily represent those of their affiliated organizations, or those of the publisher, the editors and the reviewers. Any product that may be evaluated in this article, or claim that may be made by its manufacturer, is not guaranteed or endorsed by the publisher.
